# Intratumorally specific microbial-derived lipopolysaccharide contributes to non-small cell lung cancer progression

**DOI:** 10.1080/21505594.2025.2548626

**Published:** 2025-08-16

**Authors:** Guomeng Sha, Zhengwen Wu, Biao Wang, Yi Ding, Zhaohua Xiao, Wenhao Zhang, Jie Zhou, Yongjia Zhou, Guanhong Ji, Zhongxian Tian, Weiquan Zhang, Xiaogang Zhao

**Affiliations:** a Department of Thoracic Surgery, The Second Hospital of Shandong University, Jinan, People’s Republic of China; b Key Laboratory of Precision Diagnosis and Treatment of Lung Tumors in Shandong Provincial Medicine and Health, Shandong University, Jinan, China; c Key Laboratory of Basic Research and Clinical Transformation of Thoracic Tumors in Shandong Provincial Colleges and Universities, Shandong University, Jinan, China

**Keywords:** Intratumoural microbiota dysbiosis, non-small cell lung cancer, Escherichia-shigella, *Unclassified_f_enterobacteriaceae*, lipopolysaccharide

## Abstract

As an emerging component of the tumor microenvironment (TME), the intratumoural microbiota imperceptibly influences the progression of various human malignancies. However, the critical intratumoural microbiota and its role in non-small cell lung cancer (NSCLC) progression have not been fully elucidated. Here, we used high-throughput sequencing and clinical samples analysis to identify the relationship between intratumoural bacteria and NSCLC progression. The results showed that significant abnormalities in the intratumoural microbiota of NSCLC. Specifically, the relative abundance of gram-negative bacteria in tumor was significantly increased, and network analysis revealed that *Escherichia-Shigella* and *unclassified_f__Enterobacteriaceae*, which have strong abilities to synthesize the bacterial toxin LPS, significantly promoted tumor proliferation. Mechanistically, we found that *Escherichia-Shigella-* and *unclassified_f__Enterobacteriaceae*-derived LPS activated the TLR4-mTOR-NF-κB-IL-6 axis to facilitate NSCLC cell proliferation, whereas rapamycin effectively delayed LPS-induced tumor cell proliferation in vitro and in vivo functional experiments. Receiver operating characteristic curves revealed that the combination of intratumoural bacterial concentration, *Escherichia-Shigella* abundance, *unclassified_f__Enterobacteriaceae* abundance, and LPS content had greater diagnostic validity in predicting the probability of NSCLC, and the detection of these factors in blood has potential for using the non-invasive diagnosis of NSCLC. Overall, this study revealed the mechanism by which LPS from specific bacteria in TME promoted tumor development, providing new strategies for NSCLC treatment and diagnosis from a microbial perspective.

## Introduction

Lung cancer is one of the most commonly diagnosed cancers and the leading cause of cancer-related death worldwide. According to statistics, approximately 2.0 million people are newly diagnosed and 1.8 million people die of lung cancer per year; these numbers are increasing and cause considerable challenges to healthcare systems [1]. Non-small cell lung cancer (NSCLC) accounts for 80–85% of lung cancer cases [2]. Despite the encouraging results of surgery, targeted therapy, and immunotherapy in recent years, the overall cure and survival rates for NSCLC remain low [3,4]. In addition, the use of chest computed tomography or needle biopsy to diagnose NSCLC has limitations, such as false negative results, radiation exposure and trauma to patients [5]. Therefore, exploring the mechanisms underlying NSCLC progression and identifying non-invasive diagnostic biomarkers and potential therapeutic targets are essential for improving patient outcomes.

The tumor microenvironment (TME) plays a prominent role in the response of various tumors, and the TME includes the surrounding vasculature, extracellular matrix, fibroblasts, immune cells, and the intratumoural microbiota [6,7]. Intratumoural microbiota can remodel the TME and stimulate tumor cells to alter relevant matrix components, thus playing essential roles in cancer diagnosis, development, and treatment. Recent studies have shown that the intratumoural microbiota may participate in tumor onset, progression, and prognosis through mechanisms such as the induction of DNA damage, activation of oncogenic pathways, and disruption of homeostasis between intratumoural bacteria and the host immune system [8]. For example, the intratumoural bacterium *Escherichia coli* produces colibactin, which is a polyketide-peptide genotoxin that can cause double-stranded DNA breaks and promote genomic instability, thereby leading to colorectal cancer development [9]. Moreover, tumor-colonizing *Pseudoalteromonas elyakovii* promotes breast cancer onset and progression by affecting the metabolic reprogramming of TME [10]. In a mouse CRC model, tumor-associated myeloid cell-derived IL-23 may be activated by tumor-penetrating microbes, thereby inducing inflammation and ultimately driving tumor growth [11].

Although many studies have revealed the relationship between intratumoural bacteria and tumors, the role of intratumoural bacteria in NSCLC and how they mediate tumor development remains elusive. Several studies have only characterized the microbial community of chronic obstructive pulmonary disease (COPD) or cystic fibrosis and the relationship of the bacterial community with clinical features of the lung microflora [12–14]. The latest report indicated that *Peptococcus* was associated with NSCLC prognosis, but these results have not been verified in vivo or in vitro and lack mechanistic exploration [15]. Therefore, an in-depth exploration of novel bacteria that are related to NSCLC development, as well as the underlying molecular mechanism, is needed, and such studies may lead to new diagnostic and therapeutic strategies to improve the survival of NSCLC patients.

Pathogenic bacteria produce large amounts of bacterial toxins, such as the bacterial endotoxins lipopolysaccharide (LPS), colibactin, and cytolethal, and these toxins contribute to DNA instability and increase inflammatory factor levels in the TME [16]. A persistent chronic inflammatory microenvironment can accelerate disease development [17,18]. It has been shown that LPS can activate macrophages in colon cancer, increase inflammation in the colonic epithelium, and induce pro-tumorigenic protein expression in transformed cells, thereby altering the tumor immune microenvironment [19]. LPS-stimulated bone marrow-derived dendritic cells can inhibit tumor growth efficiency by reducing the number of immunosuppressive cells in breast cancer [20]. LPS activates the inflammatory response by binding to Toll-like receptor family members, thereby triggering the innate immune inflammatory response. Studies have shown that gut dysbiosis in prostate cancer can lead to an increase in intratumoural LPS levels and STAT3 activation to produce cytokines that promote tumor proliferation and docetaxel resistance [21]. However, the ability of intratumoural pathogenic bacteria to produce LPS and the impact of their virulence on NSCLC development is currently unknown. The study of LPS from intratumoural bacteria of NSCLC will provide more intuitive evidence for the direct involvement of virulence factors in tumor development.

Here, we investigated the intratumoural bacterial profiles of NSCLC tumor tissues and adjacent nonmalignant tissues. We found that the intratumoural bacterial community of NSCLC was significantly disordered and that this dysbiosis further aggravated NSCLC progression. Intratumoural *Escherichia-Shigella-* and *Enterobacteriaceae*-derived LPS promoted NSCLC progression via the TLR4-mTOR-NF-κB-IL-6 axis, whereas the addition of the mTOR inhibitor rapamycin significantly suppressed LPS-induced tumor proliferation. In addition, the diagnostic validity of the combination of bacterial concentration, *Escherichia-Shigella* abundance, *unclassified_f__Enterobacteriaceae* abundance, and LPS content in NSCLC was greater than that of the individual factors alone. The detection of these two pathogenic bacteria, namely, *Escherichia-Shigella* and *unclassified_f__Enterobacteriaceae*, and LPS in blood samples might provide a theoretical basis for a non-invasive auxiliary diagnostic approach. This study provides new strategies for the treatment and diagnosis of NSCLC from a microbial perspective.

## Materials and methods

### Patient recruitment and sample collection

We recruited 20 patients who were diagnosed with NSCLC from May 2021 to July 2023; these patients formed the discovery cohort, and tumor and non-malignant adjacent tissues were collected from these patients. Tissue samples were obtained surgically rather than via bronchoalveolar lavage, sputum sampling, or bronchoscopic brushing, thus avoiding potential contamination by the upper respiratory tract or oral microbiota in these lung samples. Adjacent non-malignant lung tissues were obtained more than 5 cm from the tumor lesion to avoid possible effects of the tumor. Another validation cohort of 20 NSCLC patients and 20 healthy control subjects was enrolled in this study, and stool samples were collected from these participants. Peripheral blood samples were obtained from the NSCLC patients before surgery. The study inclusion criteria for NSCLC patients were as follows: (1) evidence of histologically confirmed NSCLC, (2) aged > 18 years, and (3) underwent radical surgery. The exclusion criteria included (1) diarrhea or other gastrointestinal disease, (2) treatment with any adjuvant therapies or antibiotics during the last two months, and (3) a history of other malignancies. The detailed clinical and pathological data of the discovery and validation cohorts are shown in Supplementary Tables 1 and 2, and the two cohorts of patients have similar demographics and clinical characteristics (*p* > 0.05). The samples were collected and then transferred to − 80 °C and stored until DNA extraction.

### Cell culture and reagents

The murine and human NSCLC cell lines LLC and A549 were obtained from Fu Heng Biological (Shanghai, China). LLC cells were cultured in DMEM (Corning Incorporated Cat# 10013140, New York, USA) supplemented with 1% penicillin/streptomycin (Beyotime Cat# C0211, Shanghai, China) and 10% fetal bovine serum (FBS; HyClone Cat# 04001, Logan, USA). A549 cells were cultured in Ham’s F-12K medium (BasalMedia Cat# M211106, Shanghai, China) supplemented with 10% FBS and 1% penicillin/streptomycin. The cells were incubated at 37 °C with 5% CO_2_ in a humidified atmosphere.

### DNA extraction and high-throughput sequencing of tissue and fecal samples

The microbial genomic DNA extraction and PCR processes were carried out in a UV-sterilized ultra-clean bench, and the whole process strictly followed aseptic operation and performed reagent control. Total microbial genomic DNA was extracted from 0.05 g of tissue samples with a genomic DNA purification kit (EZBioscience Cat# B0007, Roseville, USA), and DNA of total gut microbial genomic was extracted from 0.5 g samples with an SPINeasy DNA kit for feces (MPBIO Cat# 116547050, Southern California, USA) according to the manufacturer’s instructions. A DS-11 spectrophotometer (Denovix, Wilmington, USA) was used to measure the concentration and quality of the extracted DNA. The primers 338F (ACTCCTACGGGAGGCAGCAG) and 806 R (GGACTACHVGGGTWTCTAAT) were used to amplify the V3−V4 region of the 16S rRNA gene, and the product was sequenced at Novogene Co. Ltd. (Beijing, China). Standard bioinformatics producers were used to filter and control the raw data. After quality control, poorly sequenced samples were excluded, and other samples were extracted at the minimum sequencing number for subsequent analysis of microbial diversity. The raw data were deposited in the NCBI under accession number PRJNA1146577.

### Extraction and qPCR of plasma microbiome DNA

Peripheral blood samples (approximately 5 mL) were collected in EDTA tubes and processed immediately. The plasma components were separated by centrifuging the whole blood samples at 1000 × *g* for 15 min at 4 °C. Total DNA was extracted from 300 μL of plasma using a genomic DNA purification kit (EZBioscience Cat# B0007, Roseville, USA) according to the manufacturer’s instructions. Network analysis of intratumoural bacteria and clinical parameters was performed to identify microorganisms that were closely associated with tumor proliferation, and representative OTU sequences were determined based on the phylogenetic tree. Furthermore, primers that were used for qPCR were designed according to representative OTU sequences, and the qPCR system consisted of 5.0 μL SYBR mix, 0.2 μL forward primer, 0.2 μL reverse primer, 0.2 μL ROX, 3.4 μL ddH_2_O, and 1.0 μL plasma DNA. The qPCR conditions were as follows: 95 °C for 3 min, followed by 40 cycles of 95 °C for 15 s, 60 °C for 15 s, and 72 °C for 20 s. The gene expression levels were normalized to those of 16S, and the 2^−ΔΔct^ method was used to calculate relative target gene expression. The primers that were used are shown in Supplementary Table S3.

### Animal models

Female C57BL/6 mice (5 weeks old) were purchased from HFK Biotechnology (Beijing, China). The animals were housed at 25 °C with 55% humidity under a 12-h light/dark cycle and given standard water and food. LPS from *Escherichia-Shigella* (Sigma Cat# L2880, Minnesota, USA) and *Enterobacteriaceae* (Sigma Cat# L6511, Minnesota, USA) was diluted and resuspended in sterile PBS.

For an experiment on the LPS-mediated promotion of tumor proliferation, 5 × 10^5^ LLC cells suspended in 100 μL of PBS were subcutaneously injected into the right flanks of C57BL/6 mice after one week of acclimatization. When the tumors were approximately 2 mm in length, the mice were randomly divided into three groups, with five mice in each group: (1) the control group, in which mice were injected with 50 μL of PBS around the tumor; (2) the LPS-L group, in which mice were intratumorally injected with 0.5 mg/kg (50 μL) of an equal mixture of *Escherichia-Shigella-* and *Enterobacteriaceae*-derived LPS; and (3) the LPS-H group, in which mice were intratumorally injected with 1.0 mg/kg (50 μL) of an equal mixture of *Escherichia-Shigella-* and *Enterobacteriaceae*-derived LPS. The LPS dose was determined as previously described and administered twice weekly [22,23]. The size of the tumor was measured every two days via a digital caliper, and the volume of the tumor was calculated with the formula V = (length × width^2^)/2. All mice were euthanized on day 22 after the injection of tumor cells.

For the experiment in which rapamycin (MedChemExpress Cat# AY-22989, New Jersey, USA) was used to inhibit LPS-induced tumor proliferation, 7 × 10^5^ LLC cells were subcutaneously injected into C57BL/6 mice. When the tumors were approximately 2 mm in length, the mice were subsequently divided into three groups, with five mice in each group: (1) the control group; (2) the LPS group, in which mice received a peritumoral injection of an equal mixture of *Escherichia-Shigella-* and *Enterobacteriaceae*-derived LPS (0.5 mg/kg, 50 μL); and (3) the LPS + rapamycin (LPS + rapa) group, in which mice received a peritumoral injection of an equal mixture of *Escherichia-Shigella-* and *Enterobacteriaceae*-derived LPS (0.5 mg/kg, 50 μL), followed by intratumoral injected of rapamycin the next day (1.0 mg/kg, 50 μL). The mice in the control and LPS groups were injected with an equal volume of PBS. The rapamycin dose was determined as previously described [24]. On day 22, all the mice were sacrificed, and the tumors were removed and weighed immediately.

### RNA sequencing and data analysis

Three biological replicates from the tumor tissues of mice in the control, LPS-L, and LPS-H groups were randomly selected for RNA-seq library and RNA-seq analysis. After homogenization of the tissue samples, total RNA was extracted with TRNzol Universal Reagent (TIANGEN Cat# A0407A01, Beijing, China). An RNA Nano 6000 Assay Kit (Agilent Technologies, California, USA) was used to assess RNA integrity. For each sample, equal amounts of RNA were used to construct the RNA-seq library, and the samples were sent to Novogene Co. Ltd. (Beijing, China) for RNA-seq. Genes with a log_2_ (fold change) > 1 and *p* < 0.05 were considered significantly expressed genes. Kyoto Encyclopedia of Genes and Genomes (KEGG) pathway enrichment analysis was conducted with the cluster profiler package of R. The raw data were deposited in the Gene Expression Omnibus (GEO) database under accession number GSE274614.

### Enzyme-linked immunosorbent assay (ELISA)

For tissue samples, 0.02 g of tissue was weighed and added to 500 μL of lysis buffer. The samples were subsequently homogenized with a Bioprep-24 R homogenizer (Cat# 162–15191–20080001, Hangzhou, China) and ultrasonicated for 10 min. The homogenates were subsequently centrifuged at 10,000 × *g* for 5 min to obtain the supernatants, and the LPS content was determined. For blood samples, whole blood samples were collected in EDTA-stabilized tubes and centrifuged at 1000 × *g* for 15 min to obtain plasma, and the plasma LPS concentration was determined. The ELISA kits for human LPS and mouse LPS were purchased from Cloud-Clone (Cat# SEB526Hu, Wuhan, China) and CUSABIO (Cat# CSB-E13066m, Wuhan, China), respectively. ELISAs were performed in accordance with the manufacturer’s instructions.

### Cell proliferation assays

Cell proliferation was examined with a Cell Counting Kit-8 (CCK-8, NCM Cat# C6005, Suzhou, China). Briefly, 2 × 10^3^ cells were seeded in 96-well plates. The experimental groups were treated with LPS (LLC: 0.5 μg/mL of LPS; A549: 1.0 μg/mL of LPS) or rapamycin (50 μM in LLC and A549 cells), while the control group was treated with an equal volume of PBS. The LPS and rapamycin concentrations were determined as previously described and as in the preliminary experiment [25]. After the cells had adhered to the well, the culture medium was discarded, and 10 μL of Cell Counting Kit-8 (CCK-8, NCM Cat# C6005, Suzhou, China) and 90 μL of fresh culture medium were added to each well. The optical density (OD) value was measured at 450 nm after 2 h, and this value was used to determine the cell viability at 0 h. Afterward, cell proliferation was measured at 24, 48, and 72 h.

### Treatment of cells with LPS and rapamycin

For the LPS treatment assay, 2 × 10^5^ cells were seeded in 6-well plates. Low and high concentrations of LPS were added to LLC (LPS-L, 0.5 μg/mL; LPS-H, 2.5 μg/mL) and A549 (LPS-L, 1.0 μg/mL; LPS-H, 5.0 μg/mL) cells. For the rapamycin treatment assay, 2 × 10^5^ cells were seeded in 6-well plates, and three treatment groups were set up: the control group, the LPS group, and the LPS+rapa group. The final concentration of LPS in the LLC and A549 cell cultures was 2.5 μg/mL and 5.0 μg/mL, respectively. The concentration of rapamycin that was used in LLC and A549 cell cultures was 50 μM. The RNA and proteins of the cells were collected after 48 h.

### Small interfering RNA

TLR4 siRNA was synthesized by Tsingke Biotech Co., Ltd. (Beijing, China), and the primer sequences for siTLR4 are shown in Supplementary Table S4. The transfection reagent siTran 2.0 (Origene Cat# TT320002, Maryland, USA) was used for siRNA transfection. Briefly, the medium was replaced with 1.0 mL of fresh medium 30–60 min before transfection. Then, 100 μL 1×transfection buffer and 2 μL of 20 μM siRNA were added to a sterile 1.5-mL Eppendorf tube, and 3 μL of siTLR 2.0 reagent was added to the diluted siRNA. The mixed solution was transferred to the cells after incubation at room temperature for 15 min.

### RNA extraction and real-time qPCR analysis

Total RNA was extracted from tumor tissues and cells via TRNzol (TIANGEN Cat# A0407A01, Beijing, China). The purity and concentration of the total RNA were quantified via a DS-11 spectrophotometer (Denovix, Wilmington, USA). Reverse transcription was performed with ABScript Neo RT Master Mix for qPCR with gDNA Remover (ABclonal Cat# RK20433, Wuhan, China). RT–qPCR was conducted in triplicate on a QuantStudio^TM^5 Real-Time PCR system (ABI, California, USA) with an ArtiCan^ATM^SYBR qPCR Mix Kit (Tsingke Cat# TSE510, Beijing, China). The primer sequences are shown in Supplementary Table S3. GAPDH was used as the endogenous control, and relative gene expression was calculated via the 2^−ΔΔct^ method.

### Western blotting

Cells and tissues were lysed with a mixture of RIPA lysis buffer (NCM Cat# WB 3050, Suzhou, China), protease inhibitor cocktail (MCE Cat# HY-K0010, New Jersey, USA), and phosphatase inhibitor (Beyotime Cat# P1082, Shanghai, China). The protein concentration was measured with a BCA detection kit (NCM Cat# 6501, Suzhou, China), and the proteins were separated via SDS–PAGE. The protein marker used in this study was the Thermo Scientific PageRuler Prestained Protein Ladder (Cat#26616, Thermo Fisher Scientific, USA). The proteins were transferred to PVDF membranes on ice, and the membranes were blocked in 5.0% non-fat milk at room temperature for 1 h. Then, the membranes were washed three times with TBST and incubated with primary antibodies overnight at 4 °C. The next day, the membranes were washed with TBST and incubated with the corresponding HRP-conjugated secondary antibodies for 1 h at room temperature. Finally, the density of each band was measured with a Tanon 4800 system (Tanon, Shanghai, China). β-Actin was used as the internal control. The primary and secondary antibodies that were used in this study are shown in Supplementary Table S5.

### Immunohistochemistry (IHC)

IHC staining of tissue samples was conducted with an IHC kit in accordance with previously described [26]. The primary antibodies that were used were antibodies against Ki67, phospho-NF-κB p65, phospho-mTOR, and phospho-p70S6 kinase (Supplementary Table S5). After incubation with primary antibodies and secondary antibodies, the tissues were stained with DAB, and the nuclei were visualized with hematoxylin. A NanoZoomer S60 Digital Slice Scanning system (Hamamatsu, Japan) was subsequently used to obtain images. The results were evaluated via ImageJ software on the basis of the mean integrated optical density.

### Statistical analysis

All the data were repeated three times, and the error bars indicate the standard deviation of each replicate. The original data were processed and calculated using Microsoft Excel 2020 (Microsoft, Washington, USA). The line plots and bar charts were generated, and significant difference analyses were performed with GraphPad Prism 9.5 (GraphPad Software Inc., La Jolla, USA). The databases that were used in the microbial diversity analysis were SILVA 138/16S and the human oral microbiome database (HOMD). The Shapiro–Wilk test was used for normality and lognormality tests. Student’s t-test was used if the data conformed to a normal distribution between two groups; otherwise, the data were analyzed via non-parametric Mann–Whitney and Wilcoxon rank tests. One-way or two-way ANOVA was used to compare the differences among the three groups. Heatmaps were generated with the vegan package of R 4.0. Network analysis was conducted with R 4.0 and visualized with Gephi, and *p* < 0.05 was considered to indicate a statistically significant difference. The receiver operating characteristic (ROC) curves were generated using GraphPad Prism 9.5. The area under the curve (AUC) with 95% confidence interval (CI) was calculated to evaluate the diagnostic accuracy of the detected markers in predicting NSCLC. For each marker, sensitivity, specificity, and *p* value were reported. Statistical significance of AUC values was assessed with the permutation test, and all ROC analyses adhered to consistent diagnostic standards.

## Results

### Different microbial profiles in NSCLC tumor and non-malignant tissues

**Figure 1.**
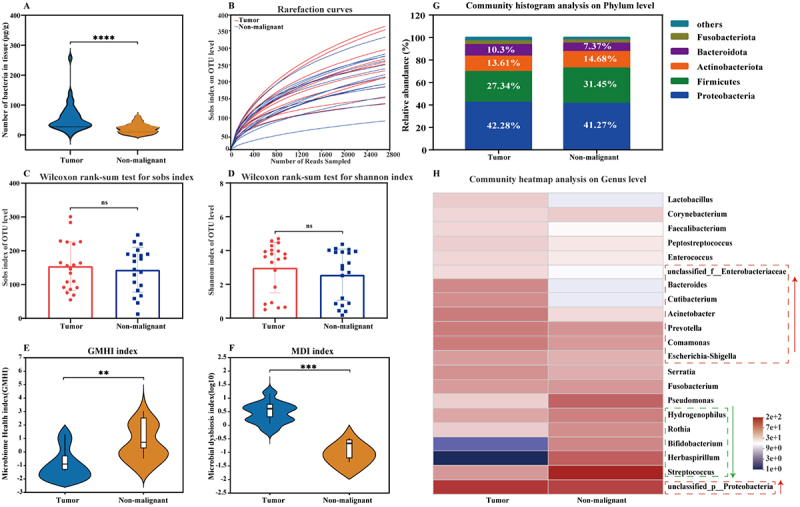
Different microbial profiles in NSCLC tumor and non-malignant tissues. (A) total bacterial DNA content in tumor and non-malignant tissues (*n* = 20). (B) rarefaction curves of the bacterial community in the tumor and non-malignant groups according to the sobs index. (C) and (D) show the Wilcoxon rank-sum test for the Sobs and Shannon indices at the OTU level in the tumor and non-malignant groups, respectively. (E) and (F) represent the differences in the microbiome health index and microbial dysbiosis index between the two groups, respectively. (G) composition of the bacterial community at the phylum level. (H) composition of the bacterial community at the genus level. The green arrow shows fewer bacteria in the tumor group than in the nontumor group, and the red arrows show more bacteria in the tumor group than in the nontumor group. Results are presented as mean ± SD. Statistical significance is assessed by Student’s t-test, Mann–whitney U or Wilcoxon rank test, and ns indicates no significance, * indicates *p* < 0.05, ** indicates *p* < 0.01, and *** indicates *p* < 0.001. See also Supplementary Figure S1.

To investigate the diversity and composition of bacterial communities in the TME, bacterial DNA was extracted from equal amounts of tumor and non-malignant tissues. The microbial content was significantly greater in the tumor group than in the non-malignant group (*p* < 0.0001) (Figure 1(A)). A total of 2,661 operational taxonomic units (OTUs) in all 40 tissue samples were identified by 16S rRNA sequencing. The rarefaction curves of the Sobs index at the OTU level gradually flattened as the number of sampled reads increased, revealing that the sequencing depth of the samples was sufficient for subsequent analyses (Figure 1(B)). The α-diversity based on the Wilcoxon rank-sum test for the Sobs and Shannon indices in the tumor group was greater than that in the non-malignant group, but the differences were not significant (Figure 1(C and D)). Notably, the microbiome health index was lower in the tumor group than in the non-malignant group (*p* < 0.01) (Figure 1(E)), whereas the microbial dysbiosis index in the tumor group was greater than that in the non-malignant group (*p* < 0.001) (Figure 1(F)). These results suggested that the intratumoural microbiome was disrupted in NSCLC.

To further characterize the intratumoural microbiota of NSCLC, the microbial community composition was assessed at the phylum and genus levels. At the phylum level, the relative abundances of *Proteobacteria* and *Bacteroidota* were enriched in the tumor group, whereas those of *Firmicutes* and *Actinobacteriota* were increased in the non-malignant group (Figure 1(G)). At the genus level, *Streptococcus*, *Herbaspirillum*, *Bifidobacterium*, *Rothia*, and *Hydrogenophilus* were depleted, whereas *unclassified_p__Proteobacteria*, *Prevotella*, *Comamonas*, *Escherichia-Shigella*, *unclassified_f__Enterobacteriaceae*, *Bacteroides*, and *Acinetobacter* were enriched in the tumor tissues from NSCLC patients (Figure 1(H)). Notably, all of the genera with increased abundance in the tumor group were gram-negative bacteria, and most of them were significantly more abundant in the tumor group than in the non-malignant group (*p* < 0.05). A marked increase in the relative abundance of gram-negative bacteria is considered a harmful sign because these bacteria are mostly pathogenic [27]. Therefore, the pathogenicity of bacteria in the tissues was analyzed via BugBase. The results revealed that the pathogenicity of the bacteria in tumor tissues was increased by 48% compared with that in non-malignant tissues (Supplementary Figure S1).

### Integrated microbiome–clinical parameter analysis linking Escherichia-Shigella- and unclassified_f__enterobacteriaceae-derived LPS with NSCLC cell proliferation

**Figure 2.**
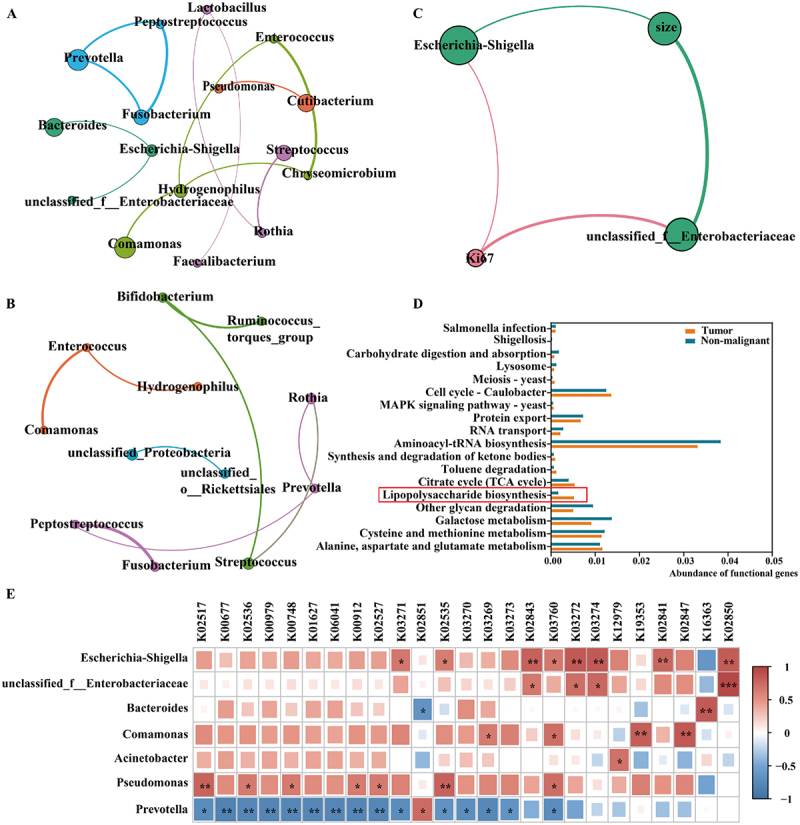
Integrated microbiome–clinical parameter analysis linking *escherichia-Shigella-* and *unclassified_f__enterobacteriaceae*-derived LPS with NSCLC cell proliferation. (A) co-occurrence patterns show the interspecies interactions among the top 20 bacteria in the tumor group. (B) co-occurrence network showing the interspecies interactions among the top 20 bacteria in the non-malignant group. (C) network analysis of the co-occurrence of bacteria genera and clinical indicators (tumor size and Ki67 expression) in tumor tissues based on Pearson correlation coefficients. (D) changes in the functional categories of bacterial communities between tumor and non-malignant tissues according to PICRUSt function prediction. (E) heatmap showing the correlation between the top 25 LPS synthesis-related functional genes and intratumoural bacteria. The linkages in the network between nodes show strong and significant correlations (r^2^ > 0.6, *p* < 0.05). Mean ± SD is used to present the results. Statistical significance is assessed, and ns indicates no significance, * indicates *p* < 0.05, ** indicates *p* < 0.01, and *** indicates *p* < 0.001. See also Supplementary figures S2 and S3.

To further localize intratumoural bacteria, which are closely associated with NSCLC cell proliferation, the interspecies interactions among the bacteria in the intratumoural microbiome were assessed. As shown in Figure 2(A and B), the tumor and non-malignant groups presented distinct modularity patterns; for example, the tumor group network had 16 points and 13 edges, and the nonmalignant group emerged 12 points and 9 edges, indicating that bacterial communities in tumor tissues had stronger network relationships. The co-occurrence network among microbial communities in the tumor tissues revealed significant positive correlations among *Bacteroides*, *Escherichia-Shigella*, and *unclassified_f__Enterobacteriaceae*, indicating a mutually beneficial relationship among the potentially harmful bacteria in tumor tissues. To further determine the relationship between intratumoural bacteria and disease, a co-occurrence network was constructed with the top 20 bacteria and the critical clinical characteristics (e.g. tumor size and Ki67 expression) of NSCLC patients. The results demonstrated that *Escherichia-Shigella* and *unclassified_f__Enterobacteriaceae* were significantly positively correlated with tumor size and Ki67 expression, respectively (Figure 2(C)).

Functional annotation was performed with PICRUSt to evaluate the metabolic pathways that are associated with the intratumoural bacteria. The results revealed that the bacteria in the tumor group had stronger lipopolysaccharide biosynthesis abilities than those in the non-malignant group (Figure 2(D)). According to the KEGG database, the bacteria in tumor tissues had almost complete LPS synthesis routes (Supplementary Figure S2). Indeed, ELISA results confirmed that the LPS levels in tumor tissues were significantly greater than those in non-malignant tissues (*p* < 0.01, Supplementary Figure S3). Moreover, the heatmap of the top 25 functional genes and intratumoural bacteria revealed that *Escherichia-Shigella* and *unclassified_f__Enterobacteriaceae*, which are involved in tumor proliferation, were significantly positively correlated with diverse genes that encode enzymes involved in LPS synthesis, such as lipopolysaccharide heptosyltransferase II (K02843), phosphate adenosyltransferase (K03272), and heptose II phosphotransferase (K02850) (Figure 2(E)). Therefore, *Escherichia-Shigella-* and *unclassified_f_Enterobacteriaceae-*derived LPS might be an important virulence factor that plays essential roles in promoting NSCLC cell proliferation.

### Escherichia-Shigella- and Enterobacteriaceae-derived LPS regulates NSCLC cell proliferation via the TLR4-mTOR-NF-κB-IL-6 pathway

To identify the specific LPS source, a phylogenetic tree was constructed with representative OTU sequences from 16S rRNA gene sequencing and *Escherichia-Shigella* and *Enterobacteriaceae* species. The representative OTU sequence of OTU9740 from *Escherichia-Shigella* was evolutionarily closest to that from the *Escherichia coli strain*, whereas the representative OTU sequence of OTU1741 from *Enterobacteriaceae* had the closest relationship with that from the *Salmonella enterica subsp. enterica serovar Typhimurium strain* (Supplementary Figure S4). Therefore, LPS derived from *Escherichia coli strain* and *Salmonella enterica strain* were selected for in vivo and in vitro studies to investigate their functional roles in NSCLC development.

**Figure 3.**
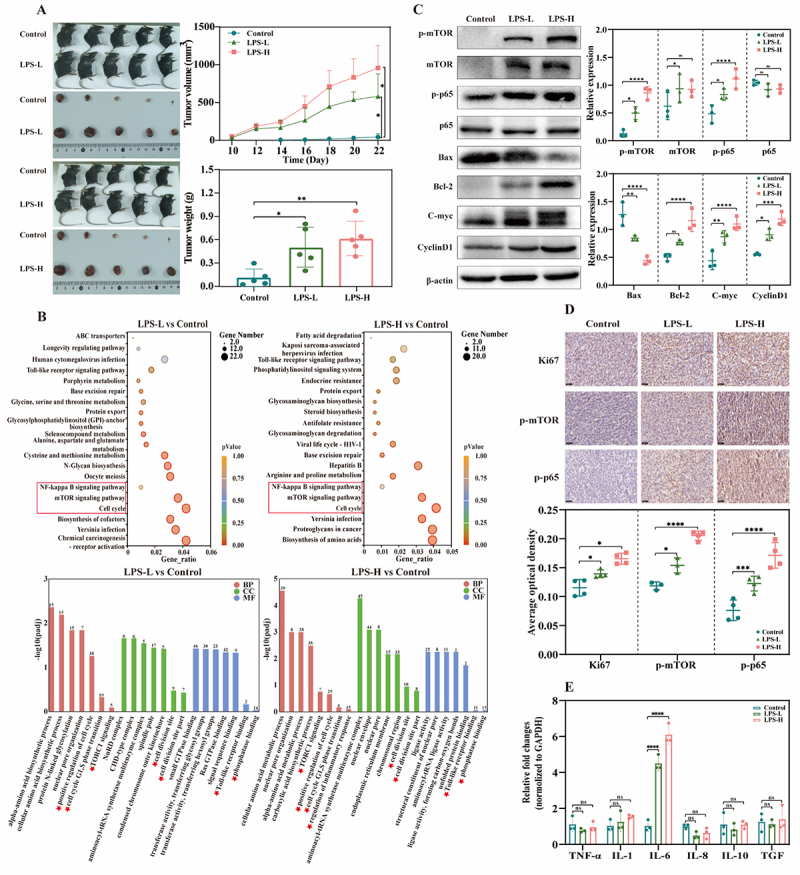
*Escherichia-Shigella-* and *Enterobacteriaceae*-derived LPS significantly promotes tumor proliferation in vivo. (A) schematic diagram of subcutaneous tumors, tumor volume (mm^3^), and tumor weight (g) in mice in the control, LPS-L, and LPS-H groups (*n* = 5). (B) KEGG and GO enrichment analysis of upregulated genes between the control and LPS-L groups and between the control and LPS-H groups. (C) western blotting analysis of relative protein expression and the statistical results of the expression of each protein in the three groups. (D) immunohistochemistry analysis of Ki67-, p-mTOR-, and p-p65-positive cells in tumor tissues from the three groups (scale bar, 25 μm). (E) transcription levels of cytokines in the three groups as determined by RT—qPCR. LPS-L represents a low dose (0.5 mg/kg) of LPS, and LPS-H represents a high dose (1.0 mg/kg) of LPS. Results are presented as mean ± SD. Statistical significance is determined by one-way or two-way ANOVA, and ns indicates no significance, * indicates *p* < 0.05, ** indicates *p* < 0.01, and *** indicates *p* < 0.001. See also Supplementary Figure S4.

LLC tumor-bearing mice were established and intratumourally injected with low or high doses of *Escherichia-Shigella-* and *Enterobacteriaceae*-derived LPS or PBS every 3 days as described in the Materials and Methods section. Compared with the control, both low and high LPS doses (LPS-L and LPS-H) significantly increased the tumor volume and weight in vivo (Figure 3(A)). To further explore the signaling pathways associated with inflammation and tumor proliferation induced by microbiota-derived LPS, transcriptome sequencing was performed on mouse tumor tissues from the control, LPS-L, and LPS-H groups. KEGG pathway analyses of the upregulated pathways revealed that the “cell cycle,” “mTOR signaling,” and “NF-kappa B (NF-κB) signaling” pathways were enriched in both the LPS-L and LPS-H groups. GO enrichment analysis revealed that the upregulated DEGs in the LPS groups were also enriched mainly in “positive regulation of cell cycle,” “TORC1 signaling,” and “phosphatase binding” (Figure 3(B)). Similarly, western blotting revealed that the essential proteins phospho-mTOR (p-mTOR) and phospho-NF-κB p65 (p-p65) (the main transcription subunit of NF-κB), as well as the cell proliferation-related proteins Bcl-2, C-myc, and CyclinD1, were markedly upregulated and that Bax was downregulated in the LPS-L and LPS-H groups (Figure 3(C)). Immunohistochemical analysis of tumor tissues revealed that in addition to p-mTOR and p-p65, the tumor proliferation index Ki67 was also increased in the LPS groups (Figure 3(D)). NF-κB signaling plays a vital role in the production of multiple inflammatory factors and facilitates inflammatory disease development [28]. The transcription levels of common inflammatory factors were subsequently measured via RT–qPCR. It was found that the relative fold change in the IL-6 levels in the LPS-L and LPS-H groups were significantly greater than that in the control group, whereas the levels of other inflammatory factors (TNF-α, IL-1, IL-8, IL-10, and TGF) did not significantly change (Figure 3(E)).

**Figure 4.**
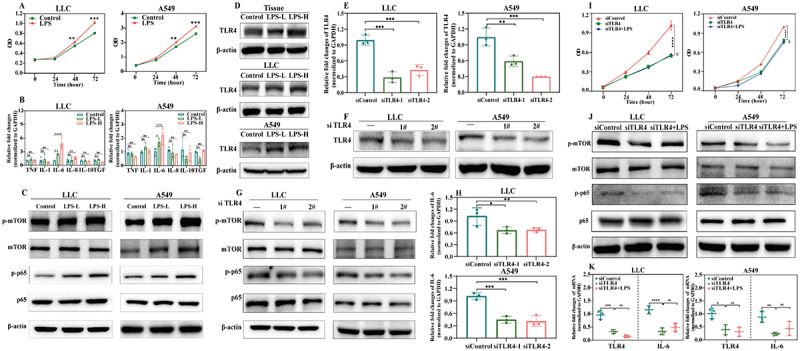
LPS significantly promotes tumor proliferation via TLR4 in vitro. (A) CCK-8 analysis of LLC and A549 cell proliferation with or without LPS for 0, 24, 48, and 72 h. (B) transcription levels of cytokines in LLC and A549 cells cultured with or without LPS for 48 h as determined via RT−qPCR. (C) western blotting analysis of relative protein expression in LLC and A549 cells treated with different concentrations of LPS for 48 h. (D) protein expression levels of TLR4 in tissues and cells and the change in TLR4 expression with increasing LPS dose. (E) RT−qPCR analysis of TLR4 mRNA levels to validate the knockdown efficiency in LLC and A549 cells. (F) protein levels of TLR4 in LLC and A549 cells were measured to show knockdown efficiency. (G) changes in downstream proteins activated by LPS after TLR4 was knocked down with two independent siRnas for 48 h in LLC and A549 cells. (H) relative fold changes in IL-6 expression in LLC and A549 cells after TLR4 knockdown with two independent siRNAs for 48 h, as determined by RT−qPCR. (I) CCK-8 analysis of LLC and A549 cell proliferation after adding exogenous LPS following the knockdown of TLR4. (J) western blotting analysis of relative protein expression in LLC and A549 cells after adding exogenous LPS following the knockdown of TLR4. (K) relative fold changes in TLR4 and IL-6 expression in LLC and A549 cells after adding exogenous LPS after the knockdown of TLR4. The results are presented as mean ± SD. Statistical significance is determined by Mann – Whitney U test, one-way or two-way ANOVA, and ns indicates no significance, * indicates *p* < 0.05, ** indicates *p* < 0.01, and *** indicates *p* < 0.001.

To elucidate the mechanism by which LPS promotes NSCLC development, the proliferation and inflammatory responses of LLC and A549 cells were evaluated. Consistently, LPS significantly stimulated NSCLC cell proliferation and increased IL-6 expression in a dose-dependent manner (Figure 4(A and B)). The p-mTOR and p-p65 protein levels also changed with increasing LPS concentration (Figure 4(C)). Notably, TLR4 is the most widely distributed TLR. It is a membrane receptor of LPS, commonly expressed in immune cells, that recognizes microbial pathogens and initiates innate immune responses [29,30]. However, whether TLR4 is expressed by NSCLC tumor cells and activated by LPS remains unclear. Therefore, the TLR4 expression level in tumor tissues and NSCLC cell lines was measured by western blotting. The results showed that TLR4 was expressed in tumor tissues, LLC cells and A549 cells and that TLR4 expression increased with increasing LPS dose (Figure 4(D)). TLR4 was downregulated to verify the biological functions of TLR4 in LLC and A549 cells, and RT–qPCR and western blotting revealed that two separate siRNAs effectively silenced TLR4 (Figure 4(E and F)). Moreover, after silencing TLR4, the key downstream proteins p-mTOR and p-p65 levels decreased, and the relative fold change in IL-6 expression was also significantly suppressed in both LLC and A549 cells (Figure 4(G and H)). Next, we added exogenous bacterial-derived LPS after TLR4 was knocked down. The results revealed that tumor cell proliferation was not significantly induced (Figure 4(I)) and that the levels of the key proteins p-mTOR and p-p65 and the level of IL-6 were not significantly increased following LPS treatment (Figure 4(J and K)). Thus, TLR4 is a vital mediator of the function of LPS in NSCLC cells. These results indicated that *Escherichia-Shigella-* and *Enterobacteriaceae*-derived LPS might be an essential virulence factor of intratumoural bacteria that promotes NSCLC proliferation via the TLR4-mTOR-NF-κB-IL-6 axis.

### Rapamycin attenuates LPS-induced NSCLC proliferation

**Figure 5.**
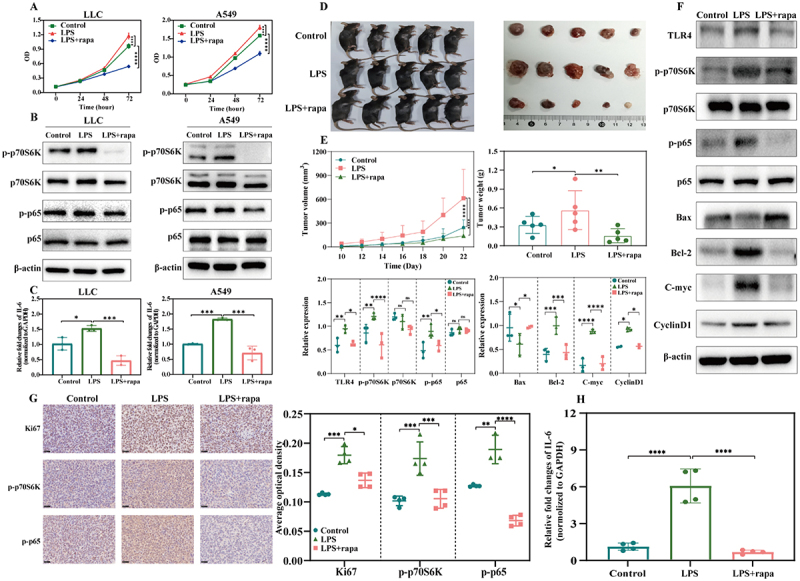
Rapamycin attenuates LPS-induced NSCLC proliferation. (A) CCK-8 analysis of LLC and A549 cell proliferation at 0, 24, 48, and 72 h in the control, LPS, and LPS+rapa groups. (B) western blotting analysis of relative protein expression in LLC and A549 cells cultured under different conditions. (C) relative fold changes in the level of IL-6 in the various treatment groups, as determined by RT–qPCR. (D) and (E) representative images of the relevant mice and tumors and comparisons of tumor volume and weight among the three groups (*n* = 5), respectively. (F) western blotting analysis of relative protein expression in the tumor tissues of mice in the control, LPS, and LPS+rapa groups. (G) immunohistochemistry of Ki67, p-p70S6K, and p-p65 in tumor tissues from the three groups (scale bar, 25 μm). (H) transcription levels of IL-6 in tumor tissues from the control, LPS, and LPS+rapa groups are measured via RT–qPCR. Results are presented as mean ± SD. Statistical significance is assessed by one-way or two-way ANOVA, and ns indicates no significance, * indicates *p* < 0.05, ** indicates *p* < 0.01, and *** indicates *p* < 0.001.

Rapamycin, which targets mTOR, was used to verify whether *Escherichia-Shigella-* and *Enterobacteriaceae*-derived LPS promoted the inflammatory response and proliferation of NSCLC mainly by activating the mTOR-NF-κB signaling pathway. A CCK-8 assay revealed that rapamycin obviously inhibited the proliferative effect of bacteria-derived LPS on NSCLC cell lines (Figure 5(A)). P70S6K is a downstream signaling molecule of mTOR that is specifically inhibited by rapamycin, and phosphorylating p70S6K (p-p70S6K) promotes cell proliferation and inhibits apoptosis [31]. Therefore, the levels of the downstream proteins p-p70S6K and p-p65 were detected via western blotting. The results showed that rapamycin significantly decreased the LPS-induced expression levels of p-p70S6K and p-p65 (Figure 5(B)). This finding also suggested that p-p65 is a downstream protein of mTOR and that its activation is influenced by p-mTOR. Moreover, IL-6 expression was upregulated in the LPS group and downregulated in the LPS+rapa group (Figure 5(C)). These results were further confirmed by in vivo experiments. Rapamycin significantly suppressed the LPS-induced increase in tumor volume and weight (Figure 5(D and E)). Western blotting analysis revealed that TLR4, p-p70S6K, p-p65, Bcl-2, C-myc, and CyclinD1 levels were increased in the LPS group but significantly decreased in the LPS+rapa group, and the Bax level showed the opposite trend (Figure 5(F)). Immunohistochemical analysis of tumor tissues revealed that the Ki67, p-p70S6K, and p-p65 levels were altered accordingly (Figure 5(G)). The IL-6 mRNA level was significantly lower in the rapamycin-treated group (Figure 5(H)). These results indicated that rapamycin could dramatically inhibit the *Escherichia-Shigella-* and *Enterobacteriaceae*-derived LPS-induced NSCLC cell proliferation by suppressing the mTOR-NF-κB axis.

### Escherichia-Shigella, Enterobacteriaceae, and LPS concentrations in blood are promising biomarkers for the non-invasive diagnosis of NSCLC

**Figure 6.**
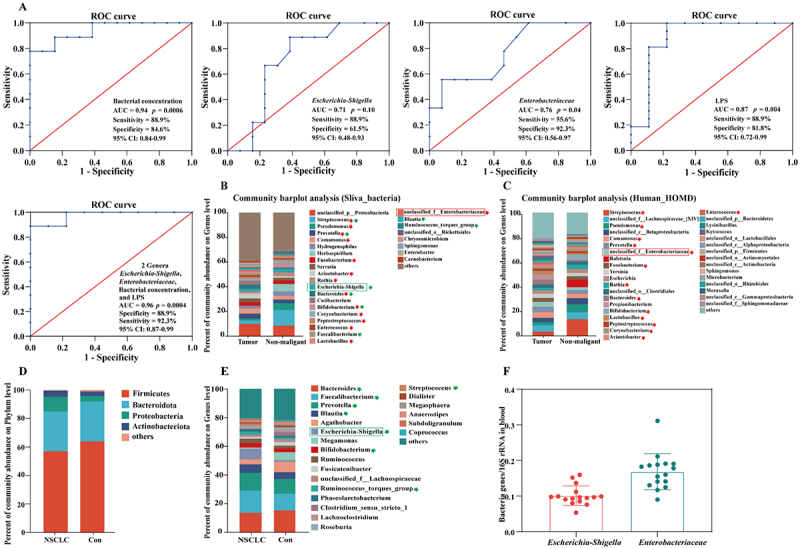
*Escherichia-Shigella* and *Enterobacteriaceae* in blood are promising biomarkers for the non-invasive diagnosis of NSCLC. (A) ROC analyses of the individual and combined scores of bacterial concentration, *Escherichia-Shigella* abundance, *unclassified_f__Enterobacteriaceae* abundance, and LPS content as predictors of NSCLC. (B) composition of community abundance at the genus level on the basis of the SILVA bacterial database. (C) the composition of community abundance at the genus level is based on the human HOMD database. (D) the composition of community abundance at the phylum level of the gut microbiota in NSCLC patients (NSCLC) and healthy controls (Con). (E) the composition of community abundance at the genus level of the gut microbiota in the NSCLC and Con groups. (F) determination of the abundance of bacteria belonging to *Escherichia-Shigella* and *unclassified_f__Enterobacteriaceae* in the blood of NSCLC patients. Genera with a relative abundance of less than 1% were classified as “others.” The red stars indicate bacteria that were shared by the NSCLC tumor microbiome and the human oral microbiome. The green stars indicate bacteria that were shared by the NSCLC tumor microbiome and the gut microbiome. See also Supplementary figures S5 and S6.

To further identify the efficacy of intratumoural bacteria in the diagnosis of NSCLC, the bacterial concentration, *Escherichia-Shigella* and *unclassified_f__Enterobacteriaceae* abundances, and LPS content were evaluated via high-throughput sequencing and ELISA. The results of the receiver operating curve analysis revealed that the area under the curve (AUC) values of bacterial concentration, *Escherichia-Shigella* abundance, *unclassified_f_Enterobacteriaceae* abundance, and LPS content were 0.94 (*p* = 0.0006, sensitivity = 88.9%, specificity = 84.6%, 95% CI: 0.84–0.99), 0.71 (*p* = 0.1, sensitivity = 88.9%, specificity = 61.5%, 95% CI: 0.48–0.93), 0.76 (*p* = 0.04, sensitivity = 55.6%, specificity = 92.3%, 95% CI: 0.56–0.97), and 0.87 (*p* = 0.004, sensitivity = 88.9%, specificity = 81.8%, 95% CI: 0.72–0.99), respectively, whereas the AUC value for the combination was 0.96 (*p* = 0.0004, sensitivity = 88.9%, specificity = 92.3%, 95% CI: 0.87–0.99) (Figure 6(A)). These findings suggested that the combination of intratumoural bacterial concentration, *Escherichia-Shigella* abundance, *unclassified_f_Enterobacteriaceae* abundance, and LPS content had greater diagnostic validity in determining the probability of NSCLC. It is worth noting that the diagnostic performance of *Escherichia-Shigella* was slightly lower compared with the other indices. Previous studies have shown that intratumoural bacteria may originate from the local normal tissue-resident microorganisms, and may be transferred between tumor and non-malignant sites through the circulatory system [32]. Therefore, the slightly lower diagnostic efficiency of *Escherichia-Shigella* in our study might be because *Escherichia-Shigella* has a certain metastasis, which is partly caused by the transfer of tumor tissues to non-malignant tissues.

Most intratumoural bacteria are obtained through surgery, but not all patients have the opportunity to undergo surgery. Therefore, there is an urgent need for more noninvasive methods for NSCLC detection and diagnosis. Thus, we began a preliminary exploration of the sources of the crucial intratumoural bacteria *Escherichia-Shigella* and *unclassified_f__Enterobacteriaceae*. Owing to the connection between the lungs and the outside of the body and the theory of the lung–gut axis [13], studies of the sources of NSCLC intratumoural bacteria have focused mainly on the oral and intestinal flora. The sequencing data were blasted against the largest HOMD database, and the results revealed that 14 types of bacterial communities in the tumor and non-malignant tissues were highly similar to those in the oral flora (Figure 6(B and C)). Among them, *unclassified_f__Enterobacteriaceae*, which is an intratumoural pathogen that promotes tumor proliferation, was also present in the oral flora. OTU 12,057 from the oral bacteria *Enterobacteriaceae* presented 96.03% sequence similarity with OTU 1741 from the NSCLC bacteria *Enterobacteriaceae*, indicating that *Enterobacteriaceae* may be inhaled orally (Supplementary Figure S5). In addition, the results of high-throughput sequencing of fecal samples from 20 NSCLC patients and 20 healthy controls revealed that *Proteobacteria* were also significantly enriched in the gut microbiota of NSCLC patients and that eight types of bacterial genera were present in both the lungs and gut (Figure 6(D and E)). In particular, pivotal intratumoural pathogenic bacteria in the NSCLC microbiota, namely, *Escherichia-Shigella*, were also present in the gut microbiota (Figure 6(B and E)). We further confirmed at the OTU level that OTU 3077 from the gut bacteria *Escherichia-Shigella* had 100% sequence similarity with OTU 9740 from the NSCLC microbiota (Supplementary Figure S5).

Previous studies have shown that the intestinal barrier is damaged in patients with tumors, after which toxic substances and bacteria in the gut might be released into circulation, allowing them to reach the tumor [21,33]. Therefore, we measured the LPS levels in the blood as an indicator of intestinal barrier integrity. The results showed that the LPS level was significantly increased in the blood of NSCLC patients (Supplementary Figure S6), suggesting that the intestinal barrier was damaged in these patients. On the basis of these findings, it is hypothesized that *Escherichia-Shigella* in the gut might reach tumors through the damaged intestinal wall via blood circulation. Therefore, it is possible that *Escherichia-Shigella* and *Enterobacteriaceae* enter the lungs from the gut and the oral cavity via the bloodstream. Hence, we extracted DNA from blood samples of NSCLC patients and performed RT–qPCR. The results revealed that pathogenic bacteria of the genera *Escherichia-Shigella* and *Enterobacteriaceae* were present in the blood of NSCLC patients (Figure 6F), suggesting that the detection of pathogenic bacteria and LPS in the blood might be a promising method for the non-invasive diagnosis of NSCLC.

## Discussion

With the development of high-throughput sequencing technology, the functional roles of the intratumoural microbiota in tumor development have gradually been revealed. However, the specific communities associated with NSCLC progression in the TME, as well as the underlying mechanisms, remain unclear. In this study, we observed intratumoural microbiota dysbiosis in NSCLC patients. In particular, the abundance of gram-negative bacteria *Escherichia-Shigella* and *unclassified_f__Enterobacteriaceae* increased, which have strong LPS synthesis capacities and are positively associated with tumor proliferation. Further investigation revealed that LPS from these bacteria promoted NSCLC proliferation via the TLR4-mTOR-NF-κB-IL-6 axis, whereas rapamycin effectively inhibited the promoting effects of bacteria-derived LPS. Moreover, the combination of intratumoural bacterial concentration, *Escherichia-Shigella* abundance, *unclassified_f_Enterobacteriaceae* abundance, and LPS content had greater diagnostic validity for NSCLC, and the ability to detect these factors in the blood allows their potential use as non-invasive biomarkers for NSCLC diagnosis in the future.

The healthy lung microbiota plays a vital role in maintaining lung homeostasis and is also considered a reflection of lung health status [34]. In the present study, through high-throughput sequencing and bioinformatics analysis, we revealed significant dysbiosis in the intratumoural microbiome in NSCLC, which was characterized by increased gram-negative bacteria. This finding suggested that increased gram-negative bacteria might affect the progression of the disease. Indeed, increasing evidence suggests that gram-negative bacteria, specifically *Proteobacteria*, participate in pathogenesis and a microbial signature of imbalance in the gut microbiota [35,36]. Among the gram-negative bacteria, *Bacteroides*, *Escherichia-Shigella*, and *unclassified_f__Enterobacteriaceae*, which belong to the *Proteobacteria* and *Bacteroidota* phyla, formed a positive co-occurrence network in tumor tissues, indicating the ecological phenomenon of mutualistic coexistence among these bacteria. In addition, *Escherichia-Shigella* and *unclassified_f_Enterobacteriaceae* were positively correlated with tumor size and the proliferation-related antigen Ki67, suggesting that *Escherichia-Shigella* and *unclassified_f_Enterobacteriaceae* might play important roles in NSCLC progression. According to previous reports, *Escherichia-Shigella* were more abundant among the gut bacteria of patients, which was valuable for diagnosing gastric and colorectal cancer [37]. The increased *Enterobacteriaceae* in the feces of patients after gastric bypass surgery could lead to DNA damage and promote inflammatory responses in the gut, increasing the risk of colorectal cancer [38]. However, a few researchers have investigated the direct effects of intratumoural *Escherichia-Shigella* and *Enterobacteriaceae* on tumors. We believe that studying the role of intratumoural *Escherichia-Shigella* and *Enterobacteriaceae* in NSCLC will provide insights into their roles in other solid tumors.

Pathogenic bacteria produce virulence factors that readily penetrate the mucus layer, causing an inflammatory response [39]. Through bioinformatics analysis, we found that the pathogenicity of bacteria in tumor tissues increased by 48% and that these bacteria had stronger LPS synthesis abilities than those in non-malignant tissues. In addition, the gram-negative bacteria *Escherichia-Shigella* and *unclassified_f_Enterobacteriaceae*, which are related to tumor proliferation, have multiple genes that encode enzymes that are involved in LPS synthesis, suggesting that the virulence factors that are produced by these bacteria could promote tumor proliferation. However, whether and how LPS derived from intratumoural pathogens promotes NSCLC progression is still unclear. Owing to the potential pathogens bacteria *Escherichia-Shigella* and *unclassified_f_Enterobacteriaceae* associated with tumor progression, we identified specific species sources of the bacterial toxin product for in vitro and in vivo experiments via evolutionary analysis. Compared with other methods, this bioinformatics analysis approach allows for the relative identification of key species origins in the microbiota based on 16S rRNA gene sequencing [21].

Moreover, we found *Escherichia-Shigella-* and *unclassified_f__Enterobacteriaceae*-derived LPS activated the mTOR-NF-κB axis, upregulated downstream proliferation genes, and elicited the production of large amounts of IL-6, contributing to the progression of NSCLC. Pathogen-induced carcinogenesis might begin by activating TLR4 in immune cells via microorganisms and their metabolites, triggering persistent inflammation and promoting the occurrence and development of tumors [7]. However, whether *Escherichia-Shigella*- and *unclassified_f__Enterobacteriaceae*-derived LPS can directly bind to TLR4 in NSCLC cells is unclear. By knocking down TLR4 on the surface of tumor cells, TLR4 was critical for *Escherichia-Shigella-* and *unclassified_f_Enterobacteriaceae*-derived LPS to promote tumor proliferation. The colonization of the TME by the microbiota is associated with bacterial virulence, and whether bacterial virulence from the microbiota directly initiates carcinogenesis is still under debate [40,41]. Our study is seemingly the first to report the direct involvement of LPS from a specific bacterial species within the tumor in promoting NSCLC proliferation. Previous research has indicated that elevated levels of IL-6 are related to the occurrence and progression of various cancers, including colorectal cancer, breast cancer, and ovarian cancer, and decreasing IL-6 might contribute to reducing the risk of cancer [29,42]. Significantly, the mTOR antagonist rapamycin inhibited the proliferative effect of LPS on tumors by downregulating downstream NF-κB and the inflammatory factor IL-6 in vitro and in an in vivo murine model. These results also indicated that NF-κB was located downstream of mTOR and was regulated by p-mTOR. However, rapamycin still has limitations in clinical NSCLC patients with *Escherichia-Shigella-* and *Enterobacteriaceae*-enriched, which is due to the complexity of rapamycin in immunomodulation and the heterogeneity of NSCLC.

The origin of intratumoural bacteria has not yet been fully elucidated. Understanding the source of crucial tumor bacteria contributes to diagnosing and predicting disease progression [29]. We found that the intratumoural bacteria *Escherichia-Shigella* and *unclassified_f__Enterobacteriaceae* might come from intestinal or oral inhalation, and these findings were further confirmed at the OTU level according to sequence similarity. Our work is consistent with the work of Pragman et al., who reported that the COPD lung microbiota originates from a physiological process, such as aspiration [13]. In addition, Aykut et al. demonstrated that *Malassezia* can migrate from the gut lumen to the pancreas and promote the progression of pancreatic cancer [43]. The gut and the lung are capable of two-way communication of microorganisms and substances via the gut-lung axis. Studies have shown that gut dysbiosis can lead to the rise in *Enterobacteriaceae*, which increases the level of IL-1β in peripheral blood by activating TLR4 and transmitting inflammatory signals to the lungs, ultimately causing lung inflammation [44]. Additionally, it was found that the intestinal bacteria *Alistipes shahii*, *Alistipes finegoldii*, and *Barnesiella visceriola* were associated with long progression-free survival (PFS), whereas *Streptococcus* was related to low PFS during the NSCLC patients receiving anti-PD1 immunotherapy [45]. Bacteria that have a shorter PFS or overall survival in response to treatment by immune checkpoint inhibitors were *Rothia* and *Streptococcus* [46]. The complexity of treatment methods causes diverse changes in the gut microbiota, thereby indirectly affecting lung microbiota through the gut-lung axis. Overall, our study has demonstrated the similarity between intratumoural bacteria and those of the gut and oral cavity.

Considering that the intestinal barrier is impaired in NSCLC patients, we hypothesized that *Escherichia-Shigella* and *Enterobacteriaceae* might travel among the gut, oral cavity, and lungs via the blood. Although the concentrations of intratumoural DNA, *Escherichia-Shigella* abundance, *unclassified_f__Enterobacteriaceae* abundance, and LPS content showed a strong ability to discriminate tumor tissues from non-tumor tissues, tissue samples are generally obtained surgically to the detriment of the patient. Previous studies have shown that microbial DNA can also be detected in blood and cell-free plasma [13,47]. With rapid advances in technology and bioinformatics approaches, diagnostic methods based on the combination of the blood microbiome and imaging evidence may improve cancer detection and prognosis [48]. Therefore, we attempted to establish a noninvasive method for detecting and diagnosing NSCLC by analyzing microorganisms in the blood; we found that bacteria were present in the blood of NSCLC patients and determined the contents of two key organisms and LPS. However, the application of microorganisms and LPS in the blood for NSCLC diagnosis must be confirmed in large sample sizes and multicenter studies.

This study had several limitations. First, our findings focused on intratumoural bacteria, which cannot yet represent all microorganisms, and metagenomic sequencing could be used in the future to map the spectrum of all microbiota within NSCLC. Second, our observations warrant further study to confirm the value of noninvasive diagnostic markers in NSCLC diagnosis and to explore their roles in other tumor types. More clinical samples and prospective studies are needed in the future to assess the value of the blood microbiota and LPS in diseases. Finally, it would be interesting to enhance the attribution of LPS production by metagenomics or cultured *Escherichia-Shigella-* and *unclassified_f__Enterobacteriaceae* approaches.

In summary, we offer new observations regarding the influence of intratumoural bacterial dysbiosis on the progression of NSCLC. Intratumoural gram-negative bacteria, which are characterized by enrichment of *Escherichia-Shigella* and *unclassified_f_Enterobacteriaceae*, increased the level of intratumoural LPS, which promoted the progression of NSCLC via the TLR4-mTOR-NF-κB-IL-6 axis in vitro and in vivo. In addition, rapamycin effectively inhibited LPS-induced tumor proliferation. Moreover, the intratumoural bacterial concentration, *Escherichia-Shigella* abundance, *unclassified_f__Enterobacteriaceae* abundance, and LPS content had greater diagnostic validity for NSCLC, and the detection of these factors in blood has the potential to be combined with imaging to improve the diagnosis of NSCLC. These results provide new strategies for NSCLC treatment and diagnosis.

## Supplementary Material

Supplementary Figure S5.tif

Supplementary Figure S1.tif

Supplementary Table 1.docx

Supplementary Figure S4.tif

Supplementary material.docx

Supplementary Table 4.docx

download Supplement.pdf

Supplementary Figure S3.tif

Supplementary Table 3.docx

Supplementary Figure S6.tif

Supplementary Table 2.docx

Supplementary Table 5.docx

Supplementary Figure S2.tif

## Data Availability

The data that support the findings of this study are openly available in https://doi.org/10.6084/m9.figshare.28279844.

## Additional information

The NSCLC patients were admitted to the inpatient Department of Thoracic Surgery at the Second Hospital of Shandong University, and the matched healthy control subjects were recruited from healthy volunteers who were admitted to the health examination center affiliated with the Second Hospital of Shandong University.

## Abbreviations

NSCLC: non-small-cell lung cancer; LPS: lipopolysaccharides; TME: tumor microenvironment; COPD: chronic obstructive pulmonary disease; FBS: fetal bovine serum; KEGG: kyoto encyclopedia of genes and genomes; GEO: gene expression omnibus; CCK-8: cell counting Kit-8; OD: optical density; IHC: immunohistochemistry; OTUs: operational taxonomic units; ELISA: enzyme-linked immunosorbent assay; p-mTOR: phospho-mTOR; p-p65: phospho-NF-κB p65; NF-κB: NF-kappa B; p-p70S6K: phosphorylating P70S6K; AUC: area under the curve; HOMD: human oral microbiome database.
