# Supplementary figures and images for: Intratumorally specific microbial-derived lipopolysaccharide contributes to non-small cell lung cancer progression
Source: Virulence. 2025 Aug 16;16(1):2548626. doi: 10.1080/21505594.2025.2548626 (PMC12363524; doi:10.1080/21505594.2025.2548626)

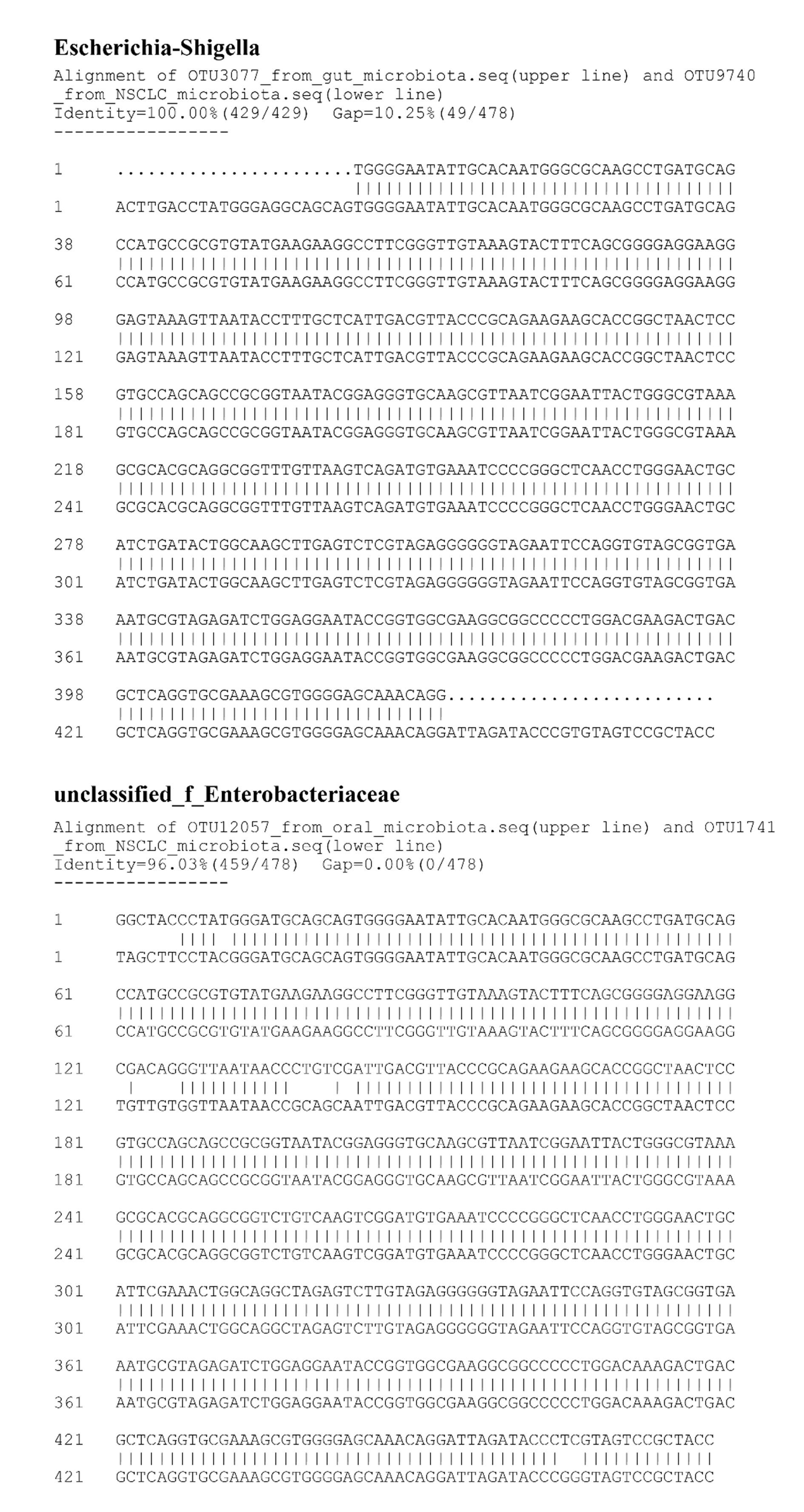

Supplement: Supplementary Figure S5.tif [file KVIR_A_2548626_SM6997.tif]

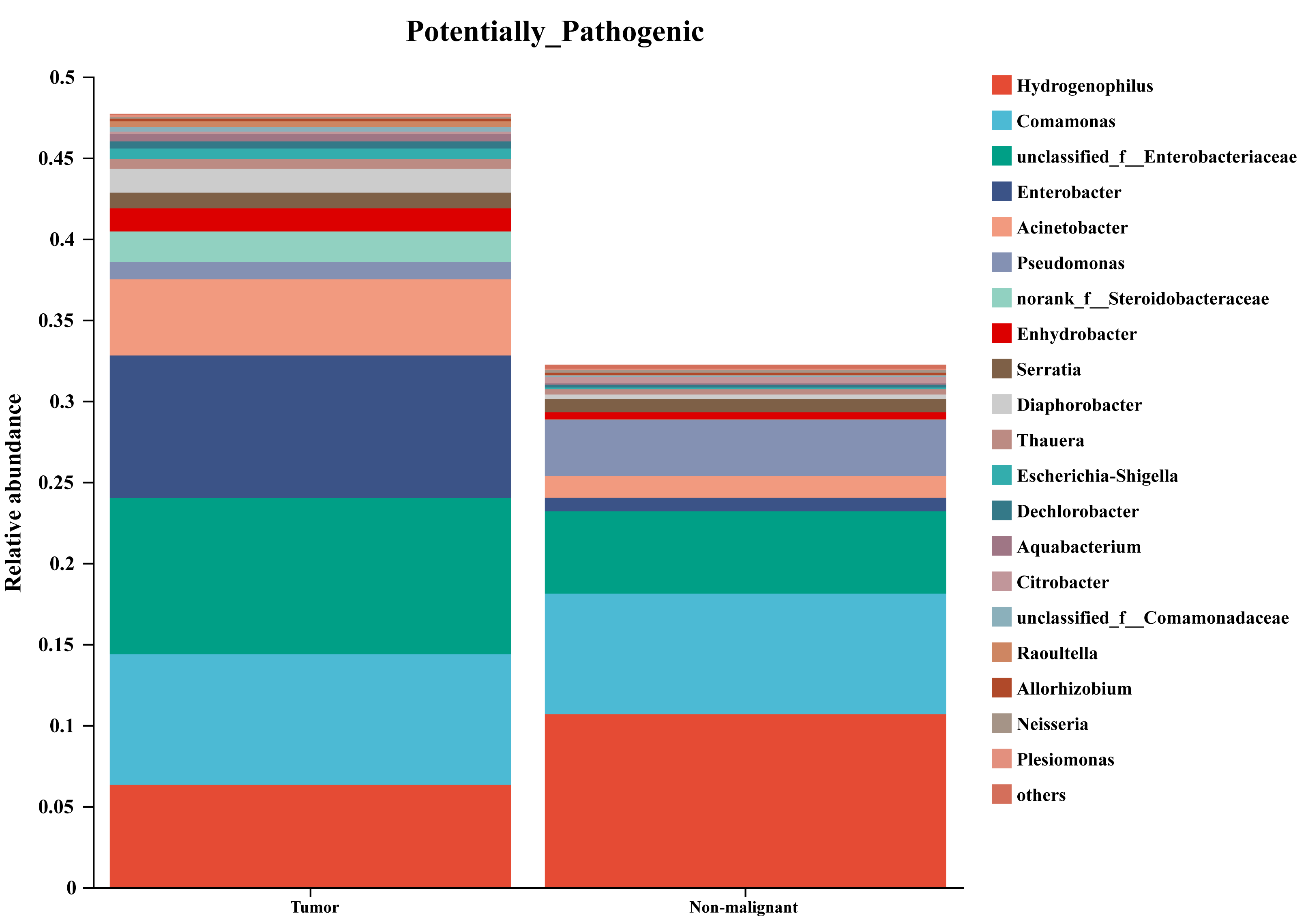

Supplement: Supplementary Figure S1.tif [file KVIR_A_2548626_SM6996.tif]

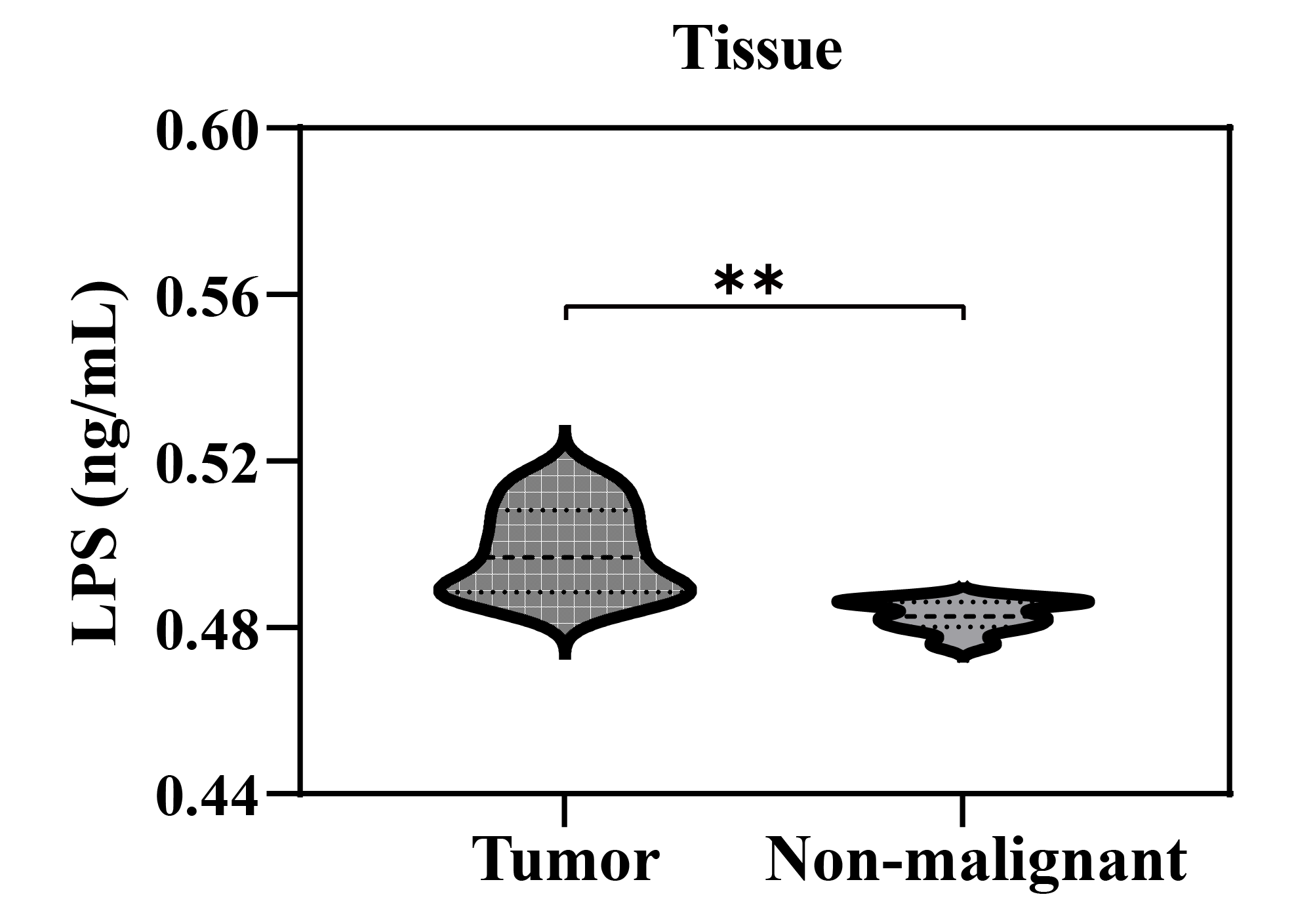

Supplement: Supplementary Figure S3.tif [file KVIR_A_2548626_SM6990.tif]

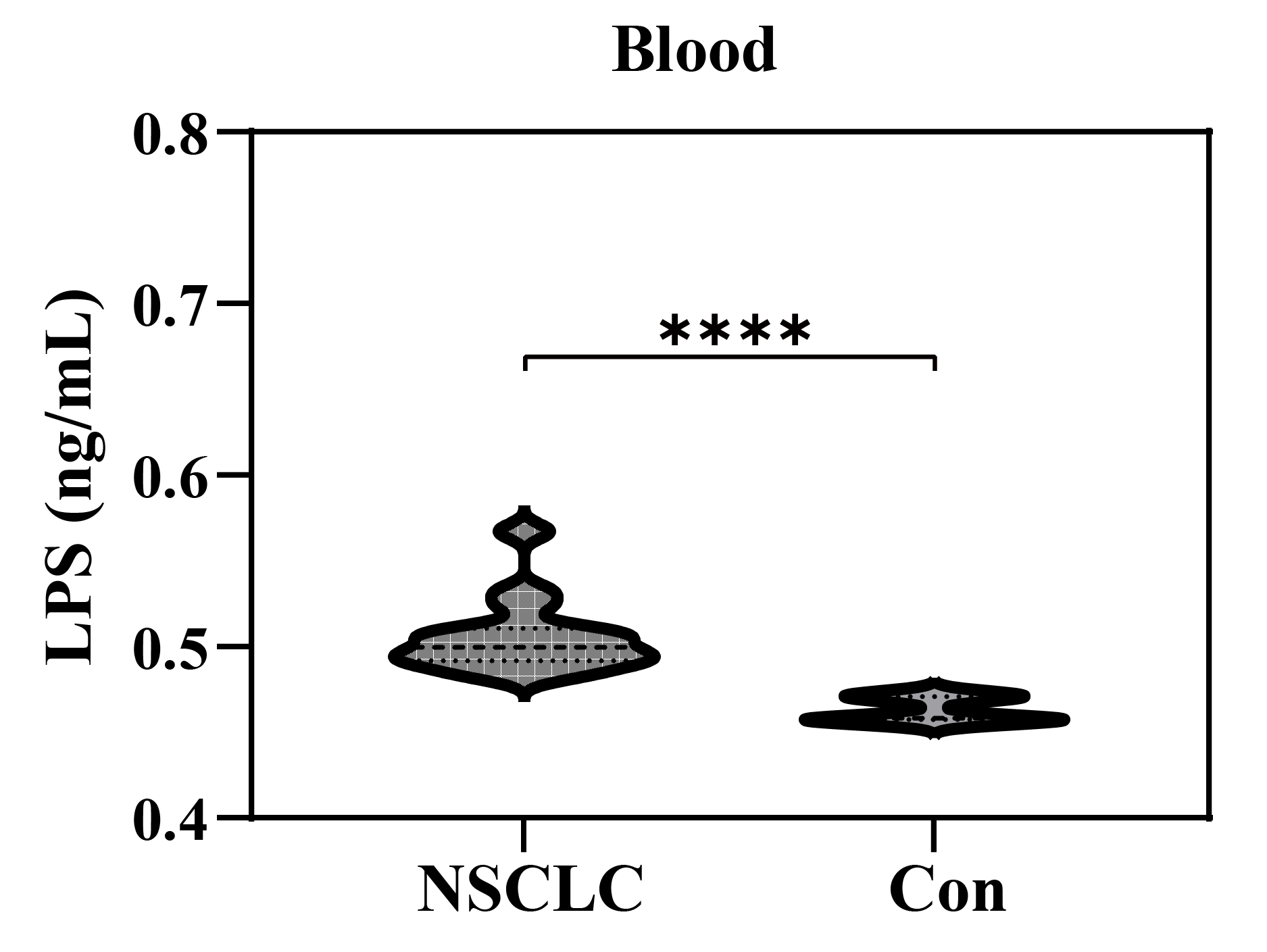

Supplement: Supplementary Figure S6.tif [file KVIR_A_2548626_SM6988.tif]

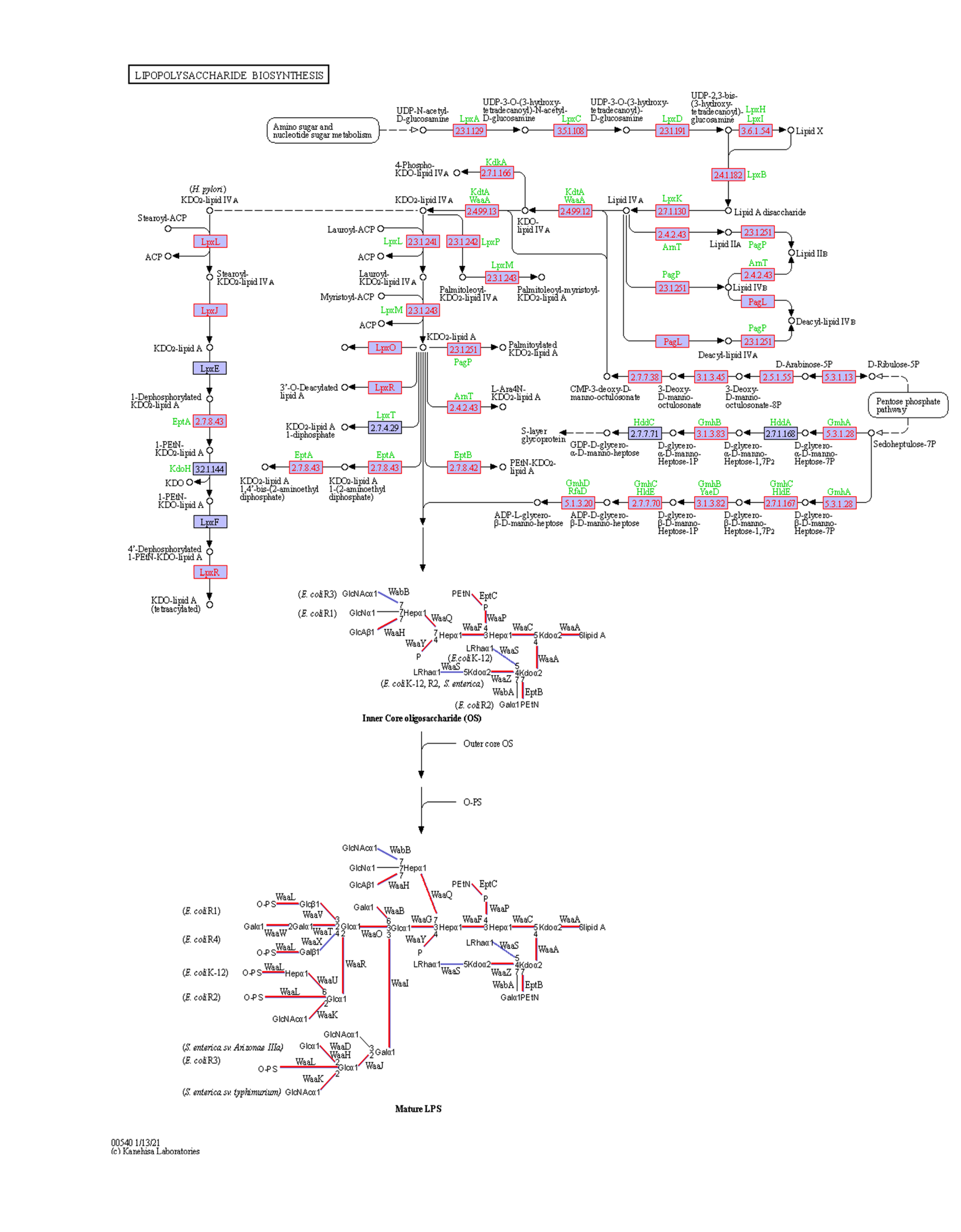

Supplement: Supplementary Figure S2.tif [file KVIR_A_2548626_SM6985.tif]
