# Supplementary material for: Intratumorally specific microbial-derived lipopolysaccharide contributes to non-small cell lung cancer progression
Source: Virulence. 2025 Aug 16;16(1):2548626. doi: 10.1080/21505594.2025.2548626 (PMC12363524; doi:10.1080/21505594.2025.2548626)
Supplement: Supplementary Table 1.docx [file KVIR_A_2548626_SM6995.docx]

**Supplementary Table 1. Demographics and clinical characteristics of the discovery cohort (n=20).**

| **Parameter** | **Median (range)** | |
| --- | --- | --- |
| **Age (years)** | 63.5 (48-80) | |
| **Sex** | **Case number (n)** | **Fraction (%)** |
| female | 9 | 45 |
| male | 11 | 55 |
| **Smoking** |  |  |
| no | 13 | 65 |
| 1-20 | 4 | 20 |
| >20 | 3 | 15 |
| **Cough** |  |  |
| no | 13 | 65 |
| yes | 7 | 35 |
| **Disease history** |  |  |
| hypertension (yes) | 4 | 20 |
| diabetes (yes) | 1 | 5 |
| coronary heart disease (yes) | 3 | 15 |
| **NSCLC histological subtype** |  |  |
| adenocarcinoma | 16 | 80 |
| squamous cell carcinoma | 4 | 20 |
| **Tumor stage** |  |  |
| I | 12 | 60 |
| II | 5 | 25 |
| III | 1 | 5 |
| IV | 2 | 10 |
| **Lesion location** |  |  |
| upper left | 4 | 20 |
| lower left | 4 | 20 |
| upper right | 9 | 45 |
| middle-lower left | 3 | 15 |
| **Tumor diameter** |  |  |
| ≤3cm | 12 | 60 |
| >3cm, ≤5cm | 7 | 35 |
| >5cm | 1 | 5 |
| **Ki67** |  |  |
| 0-10% | 9 | 45 |
| 10-50% | 9 | 45 |
| >50% | 2 | 10 |
