# Supplementary material for: Intratumorally specific microbial-derived lipopolysaccharide contributes to non-small cell lung cancer progression
Source: Virulence. 2025 Aug 16;16(1):2548626. doi: 10.1080/21505594.2025.2548626 (PMC12363524; doi:10.1080/21505594.2025.2548626)
Supplement: Supplementary material.docx [file KVIR_A_2548626_SM6993.docx]

**Intratumourally specific microbial-derived lipopolysaccharide contributes to non-small cell lung cancer progression**

Guomeng Sha,^1,2,3^ Zhengwen Wu,^1^ Biao Wang,^1^ Yi Ding,^1^ Zhaohua Xiao,^1^ Wenhao Zhang,^1^ Jie Zhou,^1^ Yongjia Zhou,^1^ Guanhong Ji,^1^ Zhongxian Tian,^1,2,3^ Weiquan Zhang,^1,2,3*^ Xiaogang Zhao^1,2,3*^

^1^Department of Thoracic Surgery, The Second Hospital of Shandong University, Jinan, 250033, People’s Republic of China

^2^Key Laboratory of Precision Diagnosis and Treatment of Lung Tumors in Shandong Provincial Medicine and Health, Shandong University, Jinan, China

^3^Key Laboratory of Basic Research and Clinical Transformation of Thoracic Tumors in Shandong Provincial Colleges and Universities, Shandong University, Jinan, China

Correspondence: Weiquan Zhang; Xiaogang Zhao, Department of Thoracic Surgery, The Second Hospital of Shandong University, Jinan, 250033, People’s Republic of China, Tel +86-17660080615; +86-053185875009, Email wqzhang91@163.com; [zhaoxiaogang@sdu.edu.cn](mailto:zhaoxiaogang@sdu.edu.cn)

Supplementary Figure S1. Composition of potentially pathogenic bacteria in tumor and non-malignant tissues.

Supplementary Figure S2. The analysis of the ability of intratumoural bacteria to produce bacterial toxin LPS using KEGG databases.

Supplementary Figure S3. LPS levels in tumor and non-malignant tissues by ELISA.

Supplementary Figure S4. Evolutionary analysis of representative OTU sequences with *Escherichia-Shigella* and *Enterobacteriaceae.*

Supplementary Figure S5. Sequences alignment of *Escherichia-Shigella* from gut and NSCLC microbiota and *unclassified_f__**Enterobacteriaceae* from oral and NSCLC microbiota.

Supplementary Figure S6. The LPS levels in blood by ELISA in NSCLC patients (NSCLC) and healthy controls (Con).
