# Supplementary material for: Intratumorally specific microbial-derived lipopolysaccharide contributes to non-small cell lung cancer progression
Source: Virulence. 2025 Aug 16;16(1):2548626. doi: 10.1080/21505594.2025.2548626 (PMC12363524; doi:10.1080/21505594.2025.2548626)
Supplement: Supplementary Table 4.docx [file KVIR_A_2548626_SM6992.docx]

**Supplementary Table 4. The sequences of siRNA used in this study.**

| **SiRNA** | **Forward Primer (5’-3’)** | **Reverse Primer (5’-3’)** |
| --- | --- | --- |
| TLR4-1-Mouse | CUUCUUCAACCAAGAACAU | AUGUUCUUGGUUGAAGAAG |
| TLR4-2-Mouse | CAAUUGACUUCAUUCAAGA | UCUUGAAUGAAGUCAAUUG |
| TLR4-1-Human | CAUUGGAUACGUUUCCUUA | UAAGGAAACGUAUCCAAUG |
| TLR4-2-Human | GAAGUUGAACGAAUGGAAU | AUUCCAUUCGUUCAACUUC |
