# Supplementary material for: Intratumorally specific microbial-derived lipopolysaccharide contributes to non-small cell lung cancer progression
Source: Virulence. 2025 Aug 16;16(1):2548626. doi: 10.1080/21505594.2025.2548626 (PMC12363524; doi:10.1080/21505594.2025.2548626)
Supplement: Supplementary Table 3.docx [file KVIR_A_2548626_SM6989.docx]

**Supplementary Table 3. The sequences of RT-qPCR primers used in this study.**

| **Target genes** | **Forward Primer (5’-3’)** | **Reverse Primer (5’-3’)** |
| --- | --- | --- |
| Mouse-TNF-α | CGTCGTAGCAAACCACCAA | GGCAGCCTTGTCCCTTGA |
| Mouse-IL-1β | TGCCACCTTTTGACAGTGATG | AAGGTCCACGGGAAAGACAC |
| Mouse-IL-6 | CTCATTCTGCTCTGGAGCCC | CAACTGGATGGAAGTCTCTTGC |
| Mouse-IL-8 | AACCTAGGCATCTTCGTCCG | TTCACCCATGGAGCATCAGG |
| Mouse-IL-10 | GGTTGCCAAGCCTTATCGGA | CACCTTGGTCTTGGAGCTTATT |
| Mouse-TGF | AAAGCAGTCAGCTGGCCTT | AGCAGTGGTAAACCTGATCCA |
| Mouse-TLR4 | TCCCTGCATAGAGGTAGTTCC | TCAAGGGGTTGAAGCTCAGA |
| Mouse-TNF-α | GCCCATGTTGTAGCAAACCC | GGAGGTTGACCTTGGTCTGG |
| Human-IL-1β | CCAAACCTCTTCGAGGCACA | GCTGCTTCAGACACTTGAGC |
| Human-IL-6 | GCCCACCGGGAACGAAAG | CGAAGGCGCTTGTGGAG |
| Human-IL-8 | CGCCAACACAGAAATTATTGTAAAG | GGTCATGAGTACAACAAACTCACT |
| Human-IL-10 | CACATCAGGGGCTTGCTCTT | GGCAACCCAGGTAACCCTTAAA |
| Human-TGF | ACAGCAACAATTCCTGGCGA | GCCGGTAGTGAACCCGTTGAT |
| Human-TLR4 | CCGTTTTATCACGGAGGTGG | GGGCTAAACTCTGGATGGGG |
| Mouse-GAPDH | CATCACTGCCACCCAGAAGACTG | ATGCCAGTGAGCTTCCCGTTCAG |
| Human-GAPDH | GTCTCCTCTGACTTCAACAGCG | ACCACCCTGTTGCTGTAGCCAA |
| *Escherichia-Shigella* | AAGCACCGGCTAACTCCGT | CCGATTAACGCTTGCACCCT |
| *Enterobacteriaceae* | AAGCACCGGCTAACTCCGT | TACGCCCAGTAATTCCGATT |
| qp-16S | GGGTTGCGCTCGTTGC | ATGGYTGTCGTCAGCTCGTG |
