# Supplementary material for: Intratumorally specific microbial-derived lipopolysaccharide contributes to non-small cell lung cancer progression
Source: Virulence. 2025 Aug 16;16(1):2548626. doi: 10.1080/21505594.2025.2548626 (PMC12363524; doi:10.1080/21505594.2025.2548626)
Supplement: Supplementary Table 2.docx [file KVIR_A_2548626_SM6987.docx]

**Supplementary Table 2. Demographics and clinical characteristics of the validation cohort.**

| **Parameter** | **NSCLC patients (n=20)** | | **Healthy control (n=20)** | |
| --- | --- | --- | --- | --- |
| **Age (years)** | 58.6 (45-72) | | 53.25 (42-69) | |
| **Sex** | Case number (n) | Fraction (%) | Case number (n) | Fraction (%) |
| female | 13 | 65 | 11 | 45 |
| male | 7 | 35 | 9 | 55 |
| **Smoking** |  |  |  |  |
| no | 16 | 80 | 16 | 80 |
| 1-20 | 0 | 0 | 0 | 0 |
| >20 | 4 | 20 | 4 | 20 |
| **Cough** |  |  |  |  |
| no | 18 | 90 | 20 | 100 |
| yes | 2 | 10 | 0 | 0 |
| **Disease history** | |  |  |  |
| hypertension (yes) | 5 | 25 | 0 | 0 |
| diabetes (yes) | 1 | 5 | 0 | 0 |
| coronary heart disease (yes) | 2 | 10 | 0 | 0 |
| **NSCLC histological subtype** | | |  |  |
| adenocarcinoma | 19 | 95 | 0 | 0 |
| squamous cell carcinoma | 1 | 5 | 0 | 0 |
| **Tumor stage** |  |  |  |  |
| I | 13 | 65 | 0 | 0 |
| II | 4 | 20 | 0 | 0 |
| III | 3 | 15 | 0 | 0 |
| **Lesion location** | |  |  |  |
| upper left | 6 | 30 | 0 | 0 |
| lower left | 5 | 25 | 0 | 0 |
| upper right | 7 | 35 | 0 | 0 |
| middle-lower left | 2 | 10 | 0 | 0 |
| **Tumor diameter** | |  |  |  |
| ≤3cm | 17 | 85 | 0 | 0 |
| >3cm, ≤5cm | 3 | 15 | 0 | 0 |
| >5cm | 0 | 0 | 0 | 0 |
| **Ki67** |  |  |  |  |
| 0-10% | 9 | 45 | 0 | 0 |
| 10-50% | 7 | 35 | 0 | 0 |
| >50% | 4 | 20 | 0 | 0 |
