# Supplementary material for: Intratumorally specific microbial-derived lipopolysaccharide contributes to non-small cell lung cancer progression
Source: Virulence. 2025 Aug 16;16(1):2548626. doi: 10.1080/21505594.2025.2548626 (PMC12363524; doi:10.1080/21505594.2025.2548626)
Supplement: Supplementary Table 5.docx [file KVIR_A_2548626_SM6986.docx]

**Supplementary Table 5. The antibodies used in the present study.**

| **Antibody** | **Cat** | **Manufacturer** | **RRID** | **Experiment** |
| --- | --- | --- | --- | --- |
| phospho-NF-κB p65 | 3033 | CST | AB_331284 | Western blot |
| NF-κB p65 | CY5034 | abways | AB_3105966 | Western blot |
| phospho-mTOR | ab109268 | abcam | AB_10888105 | Western blot |
| mTOR | CY5306 | abways | AB_3099432 | Western blot |
| phospho-p70 S6 Kinase | 9234 | CST | AB_2269803 | Western blot |
| p70 S6 Kinase | 2708 | CST | AB_390722 | Western blot |
| Bax | ab32503 | abcam | AB_725631 | Western blot |
| Bcl-2 | 12789-1-AP | proteintech | AB_2227948 | Western blot |
| C-myc | 9402 | CST | AB_2151827 | Western blot |
| CyclinD1 | 60186-1-Ig | proteintech | AB_10793718 | Western blot |
| TLR4 | sc-293072 | Santa Cruz | AB_10611320 | Western blot |
| β-actin | AC026 | ABclonal | AB_2768234 | Western blot |
| Horseradish enzyme labeled goat anti-rabbit IgG | ZB-2301 | ZSGB-BIO | AB_2747412 | Western blot |
| Horseradish enzyme labeled goat anti-mouse IgG | ZB-2305 | ZSGB-BIO | AB_2747415 | Western blot |
| Ki67 | ab92742 | ABclonal | AB_10562976 | Immunohistochemistry |
| phospho-NF-κB p65 | AF2006 | Affinity | AB_2834435 | Immunohistochemistry |
| phospho-mTOR | ab109268 | abcam | AB_10888105 | Immunohistochemistry |
| phospho-p70 S6 Kinase | 9234 | CST | AB_2269803 | Immunohistochemistry |
